# The chemopotentiation of cisplatin by the novel bioreductive drug AQ4N

**DOI:** 10.1038/sj.bjc.6600561

**Published:** 2002-11-12

**Authors:** R Gallagher, C M Hughes, M M Murray, O P Friery, L H Patterson, D G Hirst, S R McKeown

## Abstract

*British Journal of Cancer* (2002) **87**, 1339–1339. doi:10.1038/sj.bjc.6600561 www.bjcancer.com

© 2002 Cancer Research UK

**Correction to:** *British Journal of Cancer* (2001) **84**, 625–929. doi:10.1054/sj.bjc.2001.1975

## 

Unfortunately due to an error Figure 5 was reproduced incorrectly.

The correct version is printed below.

**Figure 5:**
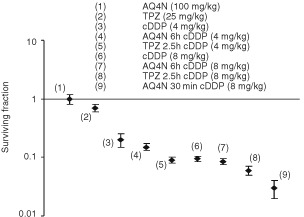
The effect of AQ4N and TPZ on the bone marrow toxicity of cDDP. BDF mice were dosed i.p. with AQ4N (100 mg/kg) 30 min or 6 h prior to cDDP (4 or 8 mg/kg). TPZ (25 mg/kg) was administered 2.5 h prior to cDDP (4 or 8 mg/kg). The survival of bone marrow cells was assessed by the spleen colony assay. Results are means±S.E. for six mice.

